# High-Resolution Network with Dynamic Convolution and Coordinate Attention for Classification of Chest X-ray Images

**DOI:** 10.3390/diagnostics13132165

**Published:** 2023-06-25

**Authors:** Qiang Li, Mingyu Chen, Jingjing Geng, Mohammed Jajere Adamu, Xin Guan

**Affiliations:** School of Microelectronics, Tianjin University, Tianjin 300072, China; liqiang@tju.edu.cn (Q.L.); 2021232008@tju.edu.cn (M.C.); gengjingjing@tju.edu.cn (J.G.)

**Keywords:** X-ray, algorithm, parallel multi-resolution network, dynamic convolution, coordinate attention

## Abstract

The development of automatic chest X-ray (CXR) disease classification algorithms is significant for diagnosing thoracic diseases. Owing to the characteristics of lesions in CXR images, including high similarity in appearance of the disease, varied sizes, and different occurrence locations, most existing convolutional neural network-based methods have insufficient feature extraction for thoracic lesions and struggle to adapt to changes in lesion size and location. To address these issues, this study proposes a high-resolution classification network with dynamic convolution and coordinate attention (HRCC-Net). In the method, this study suggests a parallel multi-resolution network in which a high-resolution branch acquires essential detailed features of the lesion and multi-resolution feature swapping and fusion to obtain multiple receptive fields to extract complicated disease features adequately. Furthermore, this study proposes dynamic convolution to enhance the network’s ability to represent multi-scale information to accommodate lesions of diverse scales. In addition, this study introduces a coordinate attention mechanism, which enables automatic focus on pathologically relevant regions and capturing the variations in lesion location. The proposed method is evaluated on ChestX-ray14 and CheXpert datasets. The average AUC (area under ROC curve) values reach 0.845 and 0.913, respectively, indicating this method’s advantages compared with the currently available methods. Meanwhile, with its specificity and sensitivity to measure the performance of medical diagnostic systems, the network can improve diagnostic efficiency while reducing the rate of misdiagnosis. The proposed algorithm has great potential for thoracic disease diagnosis and treatment.

## 1. Introduction

In clinical practice and medicine, X-ray imaging technology (X-ray), magnetic resonance imaging (MRI), and computed tomography (CT) are widely employed for disease diagnosis. Among these modalities, X-ray imaging is commonly employed for examining chest lesions due to its advantages of low radiation dose, cost-effectiveness, and ability to detect obvious lesion tissues and structures easily [1]. By utilizing the different densities and thicknesses of human tissues, X-rays create grey-scale images of chest radiographs with varying contrasts on the film [2]. However, the analysis and interpretation of the numerous chest radiographs generated worldwide heavily rely on human visual examination, which presents challenges such as the need for specialized skills, concentration, time consumption, high costs, potential operator bias, and the inability to leverage valuable information from large-scale datasets [3]. Furthermore, the shortage of radiologists proficient in reading chest radiographs poses a significant challenge to public health in many countries [4]. Hence, the development of automated algorithms for computer-aided diagnosis of chest diseases in CXR is of paramount importance.

**Figure 1:**
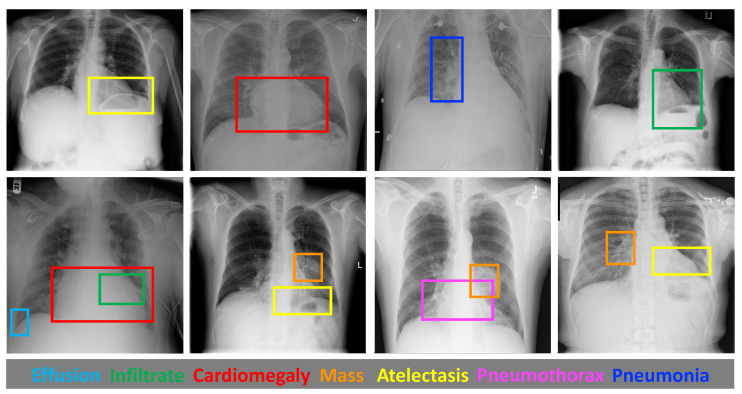
Examples of lesion areas on the ChestX-ray14 dataset. The first row shows a CXR image with a relatively small lesion area overall. The second row shows CXR images where multiple diseases are present. The disease existing in each bounding box corresponds to the pathology name in the same colour in the next row.

Numerous studies have demonstrated the effectiveness of deep learning techniques, particularly convolutional neural networks (CNNs), in medical image processing. Much research has been conducted on thoracic disease classification tasks. In recent years, deep convolutional neural networks (DCNN) have achieved impressive success in medical image classification [5,6,7]. However, despite the advancements made in this field, the automatic classification of thoracic diseases in CXR images for multi-label scenarios still requires further improvement. Firstly, the high similarity in the appearance of certain thoracic conditions on CXR images can lead to inaccuracies in distinguishing between two categories, especially for some patients with two or more pathologies. The remarkable interclass similarity observed in these images hampers the effective learning of discriminative features, thereby posing difficulties in accurately diagnosing conditions [8]. Secondly, the size of the lesions on the CXR varies considerably from one disease to another, as shown in Figure 1; for instance, cardiomegaly can encompass the entire heart, whereas effusion and mass tend to be much smaller in size. This scale variability poses difficulties for the model in accommodating and adapting to the diverse scale changes encountered while classifying different thoracic disorders. As depicted in Figure 1, the region of pneumonia can appear at various locations within the lung field, while cardiomegaly is typically located around the heart region. The presence of highly variable locations adds complexity to the task of CXR classification. Furthermore, CXR images often contain numerous areas that are irrelevant to the specific disease being diagnosed. As illustrated in Figure 1, healthy tissues constitute the majority of the image [9]. These non-disease regions provide limited diagnostic information and can impose unnecessary computational costs during analysis. Especially in exceptional cases where the lesion area is relatively small, it is necessary to exclude the interference of disease-independent regions [10]. Therefore, extracting detailed features of chest lesions and adaptively capturing variations in size and location is crucial to formulating an accurate and robust model for CXR analysis.

Currently, the prevailing frameworks for multi-label thoracic disease classification consist of serially connected networks that employ high-to-low-resolution convolutions to encode the input image into a low-resolution representation before classification. However, this approach has limitations as the extracted features may generate ambiguous mappings due to multiple convolutions, resulting in the loss of critical details. Furthermore, the aforementioned network architecture typically utilizes a single scale of the receptive domain for feature extraction in each convolutional layer. This limitation restricts the network’s ability to extract features across a wide range of spatial scales, thereby compromising its feature extraction capability to some extent. This study uses a parallel multi-resolution network as the basic network for feature extraction, which connects high-resolution to low-resolution networks in parallel. Additionally, it includes multi-resolution feature switching and fusion modules, enabling the network to capture detailed features of complex pathologies and sustain high-resolution representations. This approach is advantageous compared to traditional networks as it provides discriminative information for indistinct diseases.

To extract multi-scale information and enhance the representation of different scales in each convolutional layer, GoogLeNet [11] introduces the concept of “inception modules”. These modules utilize convolutional kernels of different sizes, enabling the extraction of features from multiple receptive domains simultaneously. This innovative approach breaks away from the traditional convolutional model and significantly improves the network’s ability to capture multi-scale information. Finally, to enhance the diversity of feature learning, a common approach is to splice and aggregate features extracted at different scales [12]. This approach can improve model performance, but it is computationally expensive. This paper uses dynamic convolution module to extract multi-scale information while not increasing many parameters and computational effort. In this case, the dynamic convolution is equivalent to conditional parametric convolution (CondConv), which can capture lesions of different sizes while improving classification accuracy.

Numerous studies [9,13] have demonstrated that the attention mechanism renders DCNN the ability to allocate more processing resources to vital information during the learning process, which makes it possible to strengthen the discriminatory power of DCNN via adapting to variances in appearance, location, and scale of thoracic abnormalities [9]. This study presents a coordinate attention (CA) module, which allows the network to focus more on focal regions while suppressing features in the picture that are irrelevant to the condition, further enhancing the classification results.

### 1.1. Challenges and Motivation

In summary, certain thoracic diseases are similar in appearance, and pathological abnormalities are difficult to discriminate with close presentation features. However, the existing state-of-the-art classification methods do not sufficiently extract the features of chest lesions and lose essential details, which leads to the recognition accuracy is not excellent. In addition, the location and size of lesion regions vary significantly for different disease categories. Such scale variability and location diversity pose difficulties for models to adapt to variations in scale and location when classifying various thoracic disorders, ultimately limiting the overall classification performance.

To address these issues, this study proposes a high-resolution classification network with dynamic convolution and coordinate attention (HRCC-Net), aiming to extract essential detailed features of the lesions as well as adapting to the variations of sizes and locations of the disease. First, this study proposes a multi-resolution network as the backbone that maintains high-resolution representations to obtain accurate, detailed lesion characteristics and thus identify subtle distinctions between different pathologies. Simultaneous multi-resolution feature swapping and fusion allow multiple receptive fields to capture rich contextual information to extract disease features adequately. Second, to enhance the network’s expression of lesions at various scales and improve the multi-scale representation of each convolutional layer, this study dynamically aggregates multiple convolutional kernels through the dynamic convolution (CondConv) module. Third, this study introduces a coordinate attention (CA) mechanism into the multi-resolution network to focus on the lesion region and extract the critical location features of the pathology by capturing location information and channel relationships. Finally, to alleviate the problem of imbalance of sample data in the dataset, this study adopts a weighted focal loss (WFL) function, which can make the network more effective in adjusting the corresponding weights according to the difficulty of disease classification.

### 1.2. Contributions

The contributions of this paper are as follows:

- This study proposes a multi-scale high-resolution network, HRCC-Net, which contains a high-resolution branch to obtain critical detail features of lesions and multi-resolution units to acquire multiple receptive fields, thus sufficiently extracting complex appearance representations of pathological abnormalities.
- This study proposes dynamic convolution (CondConv) blocks for feature extraction of diseases to acquire multi-scale information of lesions in images, adapting to lesion size variations.
- This study introduces a coordinate attention (CA) mechanism to detect the spatial location of pathological abnormalities while excluding the interference of irrelevant regions and automatically capturing changes in lesion location.

The structure of the rest of the paper is as follows. Section 2 presents the related work of the paper. Section 3 provides an in-depth description of our method. Section 4 contains the appropriate experiments and the analysis of the results. Section 5 discusses the effectiveness of our proposed network. Section 6 summarises the work of the paper.

## 2. Related Work

### 2.1. Related CNN Networks

Wang et al. [14] presented the ChestX-ray14 dataset and evaluated classical CNN architectures, namely AlexNet [15], VGGNet [16], GoogLeNet [11], and ResNet [17], to predict the presence of multiple diseases. Huang et al. [18] proposed DenseNet, a new CNN structure that outperformed the then state-of-the-art results to reach optimality in a benchmark image classification task. Rajpurkar et al. [19] suggested classifying CXR images by fine-tuning a modified DenseNet that replaced the last fully connected layer with a 14-output fully connected layer, which successfully outperformed experienced radiologists in detecting pneumonia. Chen et al. [20] proposed DualCheXNet. This network focuses on cooperative complementary learning, combining two asymmetric networks based on ResNet and DenseNet to improve the model based on the different anomalies from the original CXR to capture more discriminative features adaptively. In addition, in the diagnosis of chest X-ray images, researchers have tried to apply Transformer to images, and some works have combined CNN architecture with self-attention. Okolo et al. [21] proposed the IEViT model, built on the ViT architecture, and introduced a CNN block to classify various pathological conditions in CXR images.

### 2.2. Multi-Scale Convolution Module

Convolution kernels of different scales produce different receptive domains at each layer, allowing the extraction of multi-scale features at different levels. Multi-scale convolutional structures, such as Inception [11] and ResNext [22], have succeeded in various computer vision tasks. In such an architecture, a layer consists of multiple convolutional branches that were aggregated to compute the final output [23]. Currently, the Inception module is still a commonly used multi-scale feature extractor. Ibtehaz et al. [24] cleverly used jump connections to modify parallel-connected Inception to continuous connections and obtained different scale features. Cheng et al. [25] used an expanded Res2Net block similar to the Inception operation, which also extracted and aggregated features from a four-scale receptive domain. Xie S et al. [6] proposed ResNeXt, which was essentially a grouped convolution, where the number of groups was controlled by the number of variable bases, and the single-way convolution became a multi-way convolution with multiple branches. The dynamic convolutional layer is the mathematical equivalent of a multi-branch convolutional layer, where each branch is a single convolution, and the output is conditionally adapted to the activation of the neural network according to the input through weighting and aggregation. In this paper, the dynamic convolution module [23,26] learns to scale the activation of the output of each layer through a squeeze-excitation operation [27], which grades the input of the previous layer according to the learned attention weights [28,29,30]. Still, only one convolution needs to be computed, increasing neither the depth nor the width of the network but improving the model’s performance by attentionally aggregating multiple convolution kernels.

### 2.3. Attention Mechanism

Visual attention mechanisms enable deep learning models to learn more discriminative representations. Integrating visual attention mechanisms into deep learning has led to significant progress on many visual tasks, such as localization [31], tracking [32], visible question answering [33], and segmentation [34]. Hu et al. [27] proposed a squeeze-excitation (SE) network that adaptively recalibrates the channel feature maps by explicitly modeling the interdependencies between channels. The SE module is channel attention, which enables the network to achieve feature rescaling by learning the importance of feature channels. However, the SE module only considers channel weights and ignores spatial information. The model aims to focus on extracting pixel features of lesion locations, which requires further optimization of the attention module to enable the network to concentrate on lesion regions while reinforcing channel information. Ypsilantis et al. [35] proposed a stochastic attention-based model to determine which areas should be visually explored and to derive whether specific radiological abnormalities are present. However, only one disease, cardiomegaly, was considered in their study. Guan et al. [13] explored visual attention mechanisms. They proposed a categorical residual attention learning (CRAL) framework that aims to suppress the impairment of irrelevant classes by assigning smaller weights to feature representations, thereby addressing the problem that the recognition of one or more pathologies from CXR images is often hindered by pathologies that are not relevant to the target. However, the mechanism is guided only by the target loss function. Similar soft attention mechanisms were explored in [13,35,36] to determine which regions were more critical for classification or localization tasks. This paper uses a coordinate attention approach that can effectively capture location information and channel relationships to enhance the feature representation of the network [27], focusing information processing not only on the lesion region but also automatically locating areas of pathological abnormality.

## 3. Method

**Figure 2:**
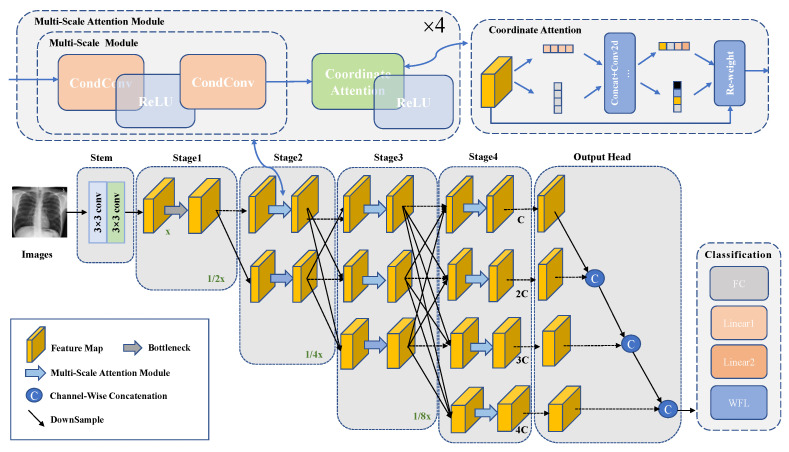
HRCC-Net network structure containing the parallel multi-resolution feature extraction network backbone and multiscale attention modules consisting of dynamic convolution (CondConv) and coordinate attention (CA). C is the number of channels for feature mapping and x is the input resolution.

This study proposes a high-resolution thoracic disease classification network (HRCC-Net) with dynamic convolution and coordinate attention to address the limitations in the classification process of existing methods for the multi-label thoracic disease. The architecture of HRCC-Net is shown in Figure 2 and Table 1. The network contains three key components: (1) Its backbone network has a parallel multi-resolution feature extraction network as its core. The network consists of multiple branches from high to low resolution, which can maintain the high resolution of the input image throughout the path, thus preserving spatial details for the identification of complicated thoracic diseases, especially those with similar feature presentation. Additionally, the duplicated multi-resolution feature exchange and fusion modules can capture rich contextual information by acquiring multiple receptive fields. (2) A multi-scale lesion feature extraction module that dynamically aggregates multiple convolutional kernels on each convolutional layer according to the relevant attention weights to improve the accuracy of feature extraction for different scale targets as much as possible without adding additional feature channels and to more fully characterize pathological images of thoracic diseases with variable sizes and slight differences in lesion textures. (3) The coordinate attention module, which simultaneously considers location information and channel relationships, enables more accurate identification and localization of the lesion’s exact location.

This paper uses two standard 3 × 3 convolutions with stride 2 for initial feature extraction of multi-labeled CXR images. Then the output feature map is fed into a parallel multi-resolution feature extraction network, which employs 4 stages in the feature extraction process. Each stage is divided into a parallel extraction feature layer and a multi-resolution fusion layer. The first stage consists of a convolution block containing 4 residual bottleneck units to ensure the quality of the original feature map. Stages 2–4 extract image features using 4 multi-scale attention modules, each containing convolutional blocks consisting of multi-scale module and coordinate attention. In the parallel extraction of features, a high-resolution to low-resolution stream is gradually added by downsampling with a 3 × 3 convolution kernel in stride 2, from a high-resolution convolution stream as the first stage, and the multi-resolution streams are connected in parallel. The multi-resolution fusion process uses a 3 × 3 deconvolution with a stride 2 for upsampling. The image’s resolution is recovered layer by layer to the dimension of the previous layer for feature fusion. Finally, the feature map is passed through the output head, followed by classification. At the same time, to improve the classification accuracy of the difficult-to-classify diseases and to achieve an overall improvement in the classification performance, this paper uses a weighted focal loss (WFL) function, which allows the network to adjust the corresponding weights more effectively according to the difficulty of the disease classification.

### 3.1. Parallel Multi-Resolution Feature Extraction Network

Inspired by the high-resolution network (HRNet) [8], this study employs the parallel multi-resolution feature extraction network as the backbone. The specific structure of the network shows in Figure 2, and the system consists of four parallel multi-resolution sub-networks, designed from top to bottom with four different resolutions of 56 × 56, 28 × 28, 14 × 14, and 7 × 7.

This network consists of 4 stages. In the first stage, the high-resolution branch is output after a convolution block containing 4 residual bottleneck units. This high-resolution branch can maintain a high-resolution representation, providing more detailed information about the area of the lesion and its location, thus identifying slight differences between lesions and between lesions and normal tissue. In contrast, for some location-sensitive lesions, the spatial location information retained by the parallel multi-resolution facilitates disease classification. In stage 2, the network structure has two resolution subnets: the original high-resolution subnet and a low-resolution subnet with half the resolution. In stages 3 and 4, a low-resolution subnet is added with half the solution and twice the number of channels of the previous branch. The multi-resolution subnetwork provides different scales of receptive fields. It addresses the problem of insufficient spatial information for high semantic information and incomplete semantic information for low feature maps rich in spatial data. It enables richer semantic information to accommodate the differences between lesions of different sizes. In addition, the repeated cross-resolution exchange and fusion module increase the resolution representation with the help of the low-resolution term allowing for richer semantic information and extraction of abundant lesion features.

### 3.2. Multi-Scale Feature Extraction Module

**Figure 3:**
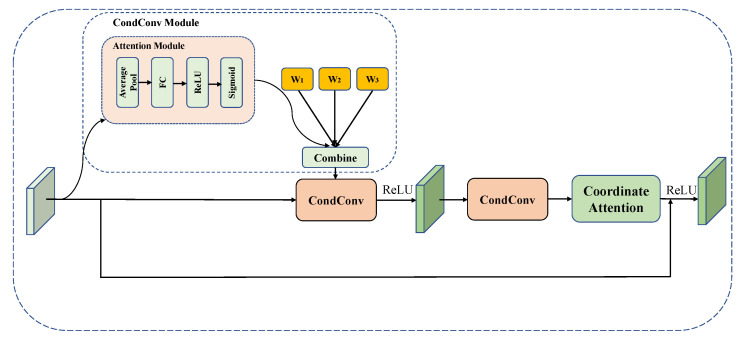
Multi-scale lesion feature extraction module structure, containing two dynamic convolution (CondConv) modules.

This study uses dynamic convolution as a multi-scale lesion feature extraction module to obtain feature information from lesions at different scales. Each convolution layer’s kernels at different scales are dynamically assigned according to the relevant attention weights to remove multi-level lesion features from various receptive fields. Because of the small size of the convolutional kernels and the fact that these kernels are aggregated in a non-linear way by attention, redundant feature information is reduced. This study uses the dynamic convolution (CondConv) module to enhance the standard convolution by inserting the CondConv module into the residual module, the exact structure of which is shown in Figure 3. After passing through the first dynamic convolution module, the feature map uses the ReLU function to activate the aggregated features, and then through another dynamic convolution, using coordinate attention to strengthen the location information, combined with the forward path, and finally through the ReLU activation to obtain the output.

This paper uses a dynamic convolution containing 3 convolution kernels, i.e., *n* = 3. The dynamic convolution output is as follows [26]:

(1) $$\mathrm{Output}\left(x\right)=\sigma (\left(\alpha_{1}\cdot W_{1}+\alpha_{2}\cdot W_{2}+\ldots +\alpha_{n}\cdot W_{n}\right)*x)$$

where each $\alpha_{i}=r_{i}\left(x\right)$ is an input-dependent learnable weight parameter, *n* is the number of convolutional kernels, σ is the activation function, and each convolutional kernel $W_{i}$ has a size equal to that of a standard convolutional kernel.

The weight coefficients $\alpha_{i}$ for each ordinary convolutional kernel are obtained through the attention module, which is shown in Figure 3. Assuming that *x* is an input to the attention module, the process can be represented as follows:

(2) $$\alpha_{i}=r_{i}\left(x\right)=\mathrm{Sigmoid}\left(\mathrm{FC}\left(\mathrm{GAP}\left(x_{i}\right)\right)\right)$$

where GAP stands for global average pooling, FC stands for fully connected and Sigmoid implements incentive operations. According to the weight coefficients σ, a dynamic convolutional kernel suitable for this input is obtained by a weighted combination of multiple convolutional kernels $W_{i}$, which can be expressed as: $\left(\alpha_{1}\cdot W_{1}+\ldots +\alpha_{n}\cdot W_{n}\right)$.

### 3.3. Lesion Location Enhancement Module

**Figure 4:**
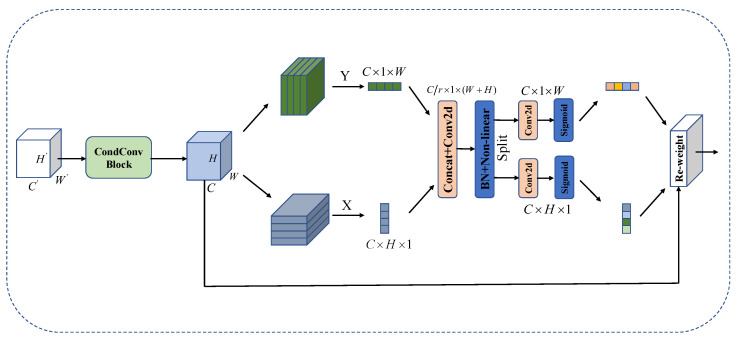
Structure of the CA mechanism.

Different pathological abnormalities commonly occur in various areas of the lung field, and to more accurately identify and localize the exact location of the lesion, excluding a large number of irrelevant disease regions in the CXR image, this paper adopts the coordinate attention (CA) mechanism. As shown in Figure 4, after putting the input feature map through the multi-scale focal feature extraction module, to alleviate the loss of location information caused by 2D global pooling, the CA block uses two 1D global pooling operations to aggregate the input features along the X and Y directions, respectively into two independent direction-aware feature maps, which allows the capture of long-distance dependencies along one spatial path while retaining precise position information along another. Then, it is put together for convolution, interacting with the information in both directions. After BN and non-linear activation functions, this feature map is split and convolved separately, focusing on horizontal and vertical directions. The two feature maps embedded with specific directional information are encoded as two attention maps. The coordinate attention weight generation process uses the features with high spatial location information generated in the coordinate embedding stage to combine the relationship between channels further to generate channel weight coefficients $g^{h}$ and $g^{w}$ in the X and Y directions to reweight the input feature images. The weights in both directions can focus on both location and channel, reinforcing both channel information and location information in the lesion area. Enabling accurate localization of the lesion, it can accurately locate the lesion and automatically capture the location changes to help the network achieve better classification.

Assume that we have the input $X=\left[x_{1},x_{2},\ldots ,x_{C}\right]\in R^{C\times H\times W}$, where C, H, and W represent the width, height, and number of channels of the feature map, respectively. The first step is the embedding of the coordinate information. Given the input *X*, then encode each channel along the horizontal and vertical coordinates using the two spatial ranges (H, 1) or (1, W) of the pooling kernel, respectively. Thus, the output of the cth channel at height h can be expressed as:

(3) $$z_{c}^{h}\left(h\right)=\frac{1}{W}\sum_{0\leq i<W}x(h,i)$$

Similarly, the output of the *c* channel of width *w* can be written as:

(4) $$z_{c}^{w}\left(w\right)=\frac{1}{H}\sum_{0\leq j<H}x(j,w)$$

In the next step of coordinate attention generation, given the aggregated feature maps generated by Equations (3) and (4), this study first concatenates and then passes them through the shared 1 × 1 convolutional transform function *F* to obtain:

(5) $$f=\delta (F\left(\left[z^{h},z^{w}\right]\right))$$

where [ , ] stands for the concentration operation, $z^{h}$ and $z^{w}$ are the 1D feature vectors output after global averaging pooling in both directions, respectively, *F* stands for the convolution operation, δ for batch normalization and hard swish activation function calculation, and *f* for the intermediate feature map output from this operation.

Then *f* is divided into two independent tensors $f^{h}$ and $f^{w}$ along different spatial dimensions, which are recovered into a tensor with the same number of channels as the input *X* by 1 × 1 convolution operation respectively, and the attention weights are mapped to the range (0, 1) by the Sigmoid function. The output can be expressed as:

(6) $$g^{h}=\sigma \left(F_{h}\left(f^{h}\right)\right)$$

(7) $$g^{w}=\sigma \left(F_{w}\left(f^{w}\right)\right)$$

where $F_{h}$ and $F_{w}$ are 1 × 1 convolution operations, σ stands for the Sigmoid activation function, and $g^{h}$ and $g^{w}$ are attention weights. The image of the output features can be expressed as:

(8) $$y_{c}\left(i,j\right)=x_{c}\left(i,j\right)\times g_{c}^{h}\left(i\right)\times g_{c}^{w}\left(j\right)$$

where *c* stands for channel, $x_{c}\left(i,j\right)$ for the input feature image and $y_{c}\left(i,j\right)$ for the output feature image.

### 3.4. Weighted Focal Loss Function

This paper uses the ChestX-ray14 dataset, where the label of each chest radiograph can be represented as a 14-dimensional vector, i.e.,

(9) $$Y=[y_{1},y_{2},\cdots ,y_{n}]$$

where *n* = 14, representing 14 diseases; $y_{i}\left(i=1,2,\cdots ,n\right)$ the valid category label for each condition, $y_{i}=1$ means having the disease and is considered positive, $y_{i}=0$ represents not having the disease and is considered harmful.

Due to the diversity of textural and hierarchical features of each disease in the ChestX-ray14 dataset, and the number of samples with class imbalance, the classification difficulty of each illness varies greatly. The loss function used in this paper is an improvement on the focal loss (FL) function to address these issues. Let $p_{i}$ represent the prediction probability of the network model for label $y_{i}=1$. Then the focal loss function for each disease is formulated as [37]:

(10) $$FL\left(p_{i}\right)=\left\{\begin{array}{cc} -\alpha {\left(1-p_{i}\right)}^{\gamma }\ln p_{i}\;\; & y_{i}=1\;\\ -\left(1-\alpha \right)p_{i}^{r}\ln\left(1-p_{i}\right)\;\; & y_{i}=0 \end{array}\right.$$

where the role of α is to balance the number of positive and negative samples for each disease, usually set to 0.25; the part of γ is to balance the number of pieces for various difficult-to-classify conditions, γ is traditionally taken as 2; the expression of the focal loss function varies with the value of y in the set [0, 1].

However, for the ChestX-ray14 dataset, the information on texture, size, and location presented on radiographs varies with each disease, resulting in a significant difference in the difficulty of classification of each disease sample label. The results of several experiments show that the classification accuracy of different diseases varies greatly. For example, the Infiltration classification is more accurate, while the hernia classification is less accurate. In the training process, to improve the classification accuracy of the difficult-to-classify diseases and thus achieve the overall classification performance, the most direct method is to increase the weight of the difficult-to-classify diseases and decrease the weight of the easier classified conditions. In addition, the larger the difference between the accuracy of the difficult-to-classify diseases and the average accuracy, the more significant the change in the corresponding weights for that disease should be and the more extensive the range of variation in its associated loss function, thus skewing the network’s computational resources towards the difficult-to-classify conditions. Therefore, to make the network more effective in adjusting the corresponding weights according to the difficulty of disease classification, this paper adds weight coefficients to the FL function as the corresponding loss function for each disease and calls it the WFL function, defined as:

(11) $$WFL\left(p_{i}\right)=\frac{\frac{1}{\alpha_{i}}}{\sum_{j=1}^{N}\frac{1}{\alpha_{j}}}FL\left(p_{i}\right)=\left\{\begin{array}{cc} -\alpha \frac{\frac{1}{\alpha_{i}}}{\sum_{j=1}^{N}\frac{1}{\alpha_{j}}}{(1-p_{i})}^{\gamma }\ln p_{i}\;\; & y_{i}=1\\ -\left(1-\alpha \right)\frac{\frac{1}{\alpha_{i}}}{\sum_{j=1}^{N}\frac{1}{\alpha_{j}}}p_{i}^{\gamma }\ln\left(1-p_{i}\right)\;\; & y_{i}=0 \end{array}\right.$$

where i,j=1,2,⋯,N is the total number of classification labels, and *N* = 14 since 14 diseases are classified in this paper; $\alpha_{j}$ stands for the accuracy of disease classification corresponding to label *j* in the last round of training.

From Equation (11), it can be seen that the loss weights are linearly related to the inverse of the disease classification accuracy, so the overall classification accuracy can be improved by increasing the proportion of losses for difficult-to-classify diseases so that the network applies more attention to them.

## 4. Experimentation and Analysis of Results

### 4.1. Datasets and PreProcessing

This experiment evaluated our method on the ChestX-ray14 dataset and used the CheXpert dataset as a complementary validation experiment to verify the classification performance for uncertainty-label-containing pathologies. The AUC scores for each pathology and the mean AUC scores for all pathologies were reported separately.

**Figure 5:**
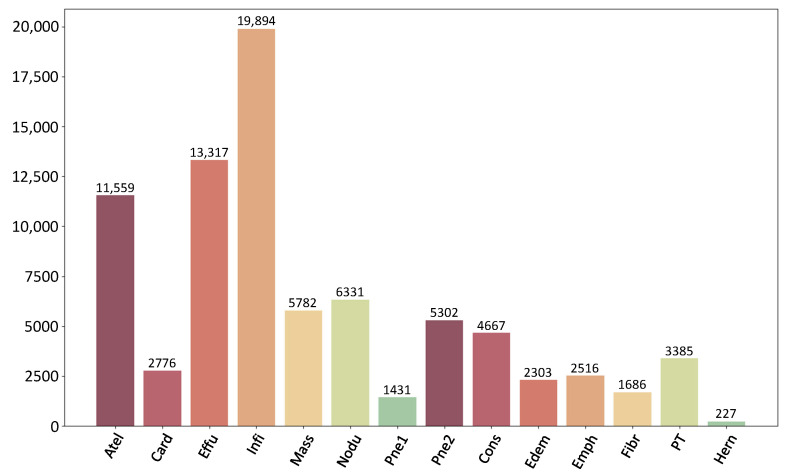
The ChestX-ray14 dataset, containing 112,120 anterior views of X-ray thoracic radiographs.

ChestX-ray14: The experiments use the large multi-label dataset ChestX-ray14 collated by the National Institutes of Health (NIH). The dataset contains a total of 112,120 front views of X-ray chest films with 14 different diseases. Of these, 60,361 images are labeled as “No Finding”; the rest are labeled as one or more chest diseases. The distribution of different conditions in the dataset is shown in Figure 5. A few disease categories have fewer samples, such as hernia, pneumonia, and fibrosis, while conditions such as infiltrates and effusions have more samples. This imbalance of sample numbers increases the difficulty of model classification. In this paper, the dataset is randomly partitioned into a training set, a test set, and a validation set in the ratio of 7:2:1 commonly to ensure that samples from the same image do not cross over in the three parts.

CheXpert: A large dataset of chest radiographs with uncertainty labels and expert comparisons. It contains 224,316 chest radiographs of 65,240 patients. A total of 14 observations are labeled in radiology reports, capturing uncertainties inherent in radiography interpretation. Samples are marked as positive, negative, or indeterminate (with different types of thoracic disease) based on the observations. The HRCC-Net is evaluated and compared for performance on the validation set segmented by [38]. It is also evaluated for five competing pathologies (atelectasis, cardiomegaly, consolidation, edema, and pleural effusion).

### 4.2. Experimental Details

This paper’s experimental code is based on Python 3.60; the server environment is Ubuntu 16.04. the experiments are based on the pytorch 1.10.1 framework for network construction and training, and the driver version is CUDA 11.3. The CPU is Intel Corei9-9900X, the graphics card is Nvidia RTX2080Ti (11GB) ×4. The training will stop when the validation loss reaches stability, using Adam optimizer for optimization. In experimental parameters, the initial learning rate Lr is a crucial hyperparameter in model training. This study experimentally compares the classification performance of the model with different initial learning rates and batch sizes. Specifically, Lr is chosen to be 0.0006, 0.0008, 0.001, 0.0012, and 0.0014 in the proposed HRCC-Net model, batch sizes of 64 and 128 are chosen, and the maximum training number is determined by the validation set. The number of training epochs is stopped when the minimum loss is achieved on the validation set and the loss is stable. Experimental results (Table 2) show when the batch size is 128, the learning rate Lr increase from 0.0006 to 0.001 mean AUC is improved, and when Lr = 0.001, the average AUC of the classification model reached an optimum of 0.845. When Lr > 0.001, the model’s performance decreases and may deviate from the optimal value. Therefore, to achieve the best classification effect, we set the initial learning rate Lr to 0.001, the batch size to 128, and the maximum number of training rounds epoch to 80.

To objectively and comprehensively evaluate the network training process, the following data enhancement techniques were applied to the training set in this study. The original chest film image in the dataset is a grayscale image of 1024 × 1024 pixels. To reduce the computational effort and to match the network model, the image is scaled to 256 × 256 pixels and converted to RGB 3-channel format. The image is randomly selected at the center and cropped to 224 × 224 pixels, and randomly flipped horizontally to achieve data enhancement. Finally, the thorax is converted to vector format and normalized to a pixel value restricted to 0–255 for readout by the network.

### 4.3. Evaluation Metrics

This paper defines thoracic disease classification as a 14-dimensional binary classification task for a multi-label classification problem, i.e., each CXR image has only two cases of positive class (containing the label) and negative class (not containing the label) for each disease label. To objectively and comprehensively evaluate the diagnostic performance of the network and to facilitate comparison with other algorithms, this paper uses the receiver operating characteristics (ROC) curve to represent the algorithm’s ability to identify each disease and calculates the area under ROC curve (AUC) values for quantitative analysis and comparison. The ROC curve is used to analyze the classification effect of the binary classification model, which outputs positive and negative classes. It can visually reflect the performance of the classifier. Its horizontal axis is the False Positive Rate (FPR); FPR stands for the probability that the classifier will misclassify a positive case in all negative samples. The vertical axis is the True Positive Rate (TPR), and TPR stands for the possibility that the classifier will correctly classify a positive point in all positive samples, calculated as [39]:

(12) $$FPR=\frac{FP}{FP+TN}$$

(13) $$TPR=\frac{TP}{TP+FN}$$

Meanwhile, the average accuracy, sensitivity, specificity and F1 are used as additional evaluation metrics to further validate the classification performance of the proposed method, which can be expressed as:

(14) $$Accuracy=\frac{TP+TN}{TP+FP+FN+TN}$$

(15) $$Sensitivity=\frac{TP}{TP+FN}$$

(16) $$Specificity=\frac{TN}{FP+TN}$$

(17) $$F1\text{-}score=\frac{2\times TP}{2\times TP+FP+FN}$$

FP is the false positive case, TN is the true negative case, TP is the true positive case, and FN is the false negative case. The curve depicts the game between the true positive rate (vertical) and the false positive rate (horizontal). Therefore, the closer the curve is to the top left corner, the better the classification performance of the algorithm, indicating that the network obtains a high actual class rate while the false positive class rate is low for the classification of samples. In the ROC curve, the function f(x) = x represents the random result, which means the most inferior performance of the classifier, so the ROC curve is generally located above the function.

The AUC value represents the area under the ROC curve. It is a probability value, taking matters in the range [0.5, 1], indicating the potential for an optimistic sample prediction to be greater than the probability of a negative prediction. Therefore, the AUC value is positively correlated with the accuracy of disease classification, and the higher the AUC value, the higher the accuracy of the classification of the corresponding disease. The average AUC value is used to evaluate the overall classification performance of the network model.

### 4.4. Results and Analysis

#### 4.4.1. Results on ChestX-ray14

**Figure 6:**
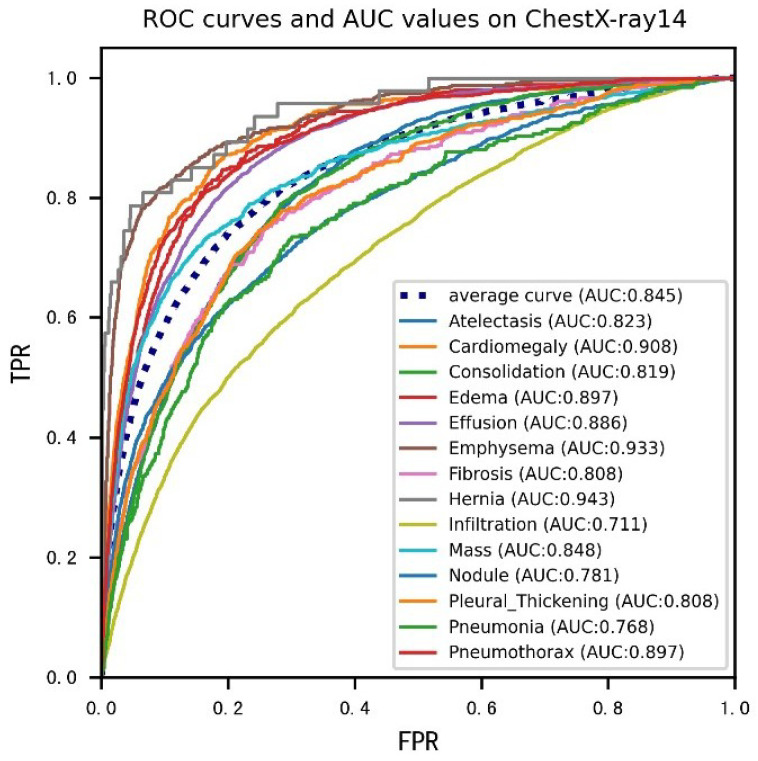
ROC curves of the HRCC-Net network for 14 pathologies, corresponding to the AUC scores in Table 3.

The ROC curves in Figure 6 depict the performance of the algorithms in this paper on the ChestX-ray14 dataset. The figure shows the ROC curve for each disease and the average ROC curve for the 14 diseases, respectively. From the definition of the ROC curve, it is clear that the point (0, 0) in the lower-left corner of the curve indicates that all samples are predicted as negative class. Point (1, 1) in the upper right corner indicates that all samples are predicted as positive classes. The point (1, 0) in the lower right corner means that all positive class samples are predicted as negative. Currently, the classification effect is the worst. The point (0, 1) in the upper left corner indicates that all samples are correctly classified, at which point the best classification is achieved.

As shown in Figure 6, all curves located above the function f(x) = x, and most curves are located in the upper left-hand corner overall, indicating that the algorithm has good overall classification performance for 14 diseases, with an average AUC of 0.845, confirming the algorithm’s effectiveness. Meanwhile, the distribution of 14 curves on the coordinate axes is denser and clustered, our algorithm can achieve a certain degree of balance in the accuracy of each disease classification.

**Figure 7:**
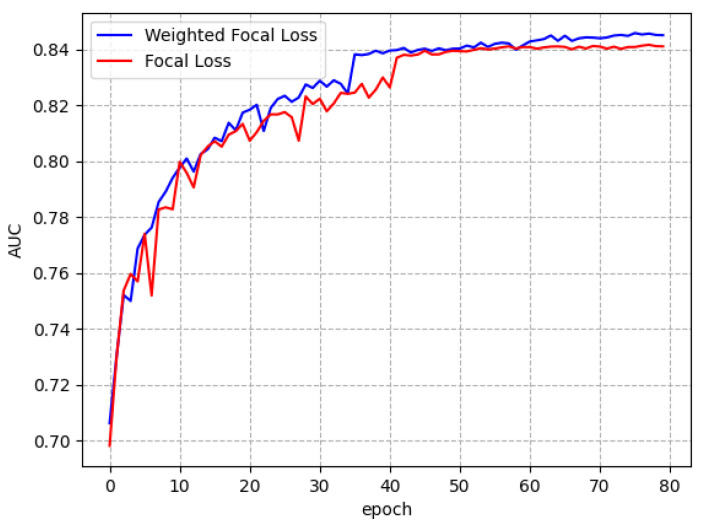
AUC values for each training epoch with different loss functions.

This study applied focal loss function and weighted focal loss function on the algorithm model proposed for experiments, respectively, as shown in Figure 7. This paper tests the average AUC values at each epoch, and stop training and testing when the loss function reached stability. The curve is plotted on the same axis, with the training epoch as the horizontal axis and the average AUC value as the vertical axis. When WFL is applied, the model curve rose smoothly, the model performance was better, and the WFL is more effective to improve the learning ability of the model. It allows the network to target and exert more attention on the more difficult-to-classify diseases, thus optimizing the overall classification performance.

To validate the performance of the proposed network, this paper compared HRCC-Net with nine recent deep-learning networks on the ChestX-ray14 dataset. The comparison networks chosen were those with better classification results so far; namely, Wang et al. [14] proposed a benchmark classification network based on ResNet-50, Yao et al. [40] suggested a weakly supervised diagnosis network, Ma et al. [41] proposed a multi attentive classification network based on ResNet-101, Guan et al. [10,13] presented a categorical residual attention learning (CRAL) framework based on ResNet and DenseNet as well as a two-branch network constructed via DenseNet-121, Wang et al. [9] proposed a three-attention learning $A^{3}$ Net model based on DenseNet-121, Ouyang et al. [42] proposed a hierarchical attention learning algorithm for weakly supervised learning, Zhu et al. [43] proposed a pixel-level classification and attention network (PCAN) with DenseNet-121 as the backbone, and Chen et al. [39] proposed a semantic similarity graph embedding (SSGE) framework. In which the multi-resolution backbone network is used as the baseline network. Table 3 gives the AUC per category and the average overall classes obtained from our model and the other nine methods. The highest used to diagnose thoracic disease in each row is highlighted in bold.

First, the performance of the baseline network was evaluated on the ChestX-ray14 dataset, and the average AUC of the multi-resolution network reaches 0.833, which is competitive compared to all other networks. Regarding each pathology, the baseline network shows an improvement of more than 2% for diseases with difficult-to-identify pathological features of effusion, pneumonia, consolidation, and edema. Notably, the AUC of effusion improves by about 4.3% from 0.840 to 0.883, and the AUC of consolidation improves by about 4.7% from 0.763 to 0.810. Cardiomegaly obtained the best classification accuracy among several methods (0.915). Other disorders, such as infiltration and hernia, are less different than Wang et al. [9]. In addition, it was observed that nodule and pleural thickening are identified with low accuracy in several methods, indicating that the network has insufficient ability to detect minor target diseases and cannot focus on disease areas. However, compared to the final model, introducing the multi-scale attention module results in a 2% improvement in the accuracy of the module.

Then, the methods in this paper are compared with those of others. Chen et al.’s method is the most advanced method at present. At the same time, the model in this paper achieves an average accuracy of 0.845 for identifying 14 diseases, which is the best among all methods. Especially the most apparent improvement compared with [14,40], with 10% and 8.4% performance improvement, respectively, and 1.2% compared to the baseline. Regarding AUC values for each disease, our model achieved the highest AUC in 10 of the 14 pathologies. Most importantly, this model is adaptable to disease with different scale sizes, all with specific enhancement, such as cardiomegaly, pneumothorax, effusion, mass. Among these pathologies, the AUC of atelectasis, effusion, pneumonia, consolidation, and edema are all improved by 3% or more compared to other methods. For some other pathologies, the improvement is less noticeable, e.g., hernia, fibrosis. In addition, the baseline network performs well on fibrosis, but the accuracy decreases after introducing the multi-scale module, which is worth exploring. This difference may be due to the number of positive samples (e.g., only 227 hernia and 1686 fibrosis samples). In summary, this model is competitive with current deep learning network networks.

#### 4.4.2. Results on CheXpert

The performance evaluation of HRCC-Net is conducted on the CheXpert dataset to assess its comprehensiveness and robustness. This paper primarily focuses on comparing the results obtained using the single-model architecture. On the CheXpert dataset, due to the uncertainty setting of the training labels, this paper explicitly merges the uncertainty labels in CheXpert with the help of two uncertainty labeling methods commonly used in multi-label classification [38]: (1) replacing all uncertainty labels with “0”; (2) replacing all uncertainty labels with “1” labels. Table 4 illustrates the experimental results, using the multi-resolution backbone network as the baseline, the average AUC scores for the five pathologies reached 0.875 and 0.879 for the “0” and “1” strategies, respectively. With HRCC-Net, both improve by more than 2% with 0.904 and 0.913, respectively. In particular, when the uncertainty label is set to “1” the mean AUC score exceeds the corresponding baseline by approximately 3.5%. When the uncertainty label is set to “0”, the performance of cardiomegaly and edema improved significantly. When set to “1”, the performance of atelectasis and edema enhanced considerably in terms of AUC score. This paper compares the method with several other ways. When the uncertainty label is set to “1”, our model performs best in the AUC and the average AUC for all pathologies except atelectasis. When set to “0”, the performance of disease classification is improved to a certain extent compared to other methods.

#### 4.4.3. Computational Consumption Analysis

In addition, computational consumption such as parameters and floating point operations (FLOPs) is also a factor that should be considered in experiments. The number of parameters and FLOPs of HRCC-Net are given in Table 5. Taking 256 × 256 input images as an example, and using multi-resolution backbone network as the baseline, the model parameters increase from 20.9 M to 23.1 M after adding the CondConv module, and the FLOPs increase from 9.71 G to 10.87 G. The model parameters and computational effort increase but not much, indicating that the CondConv module can maintain efficient inference while improving the multi-scale representation of the convolutional layer. With the addition of CA module, the FLOPs and Parameters are slightly increased. The CA module focuses on the relevant focal areas without increasing the computational volume too much. Moreover, the time used for training is different when using batch-sized training data. In our experiments, when the batch size is 64, it takes about 22 h to train the HRCC-Net network, consuming approximately 28.3 G of GPU memory. When the batch size is set to 128, the experimental device can be fully utilized, occupying 36.2 G of GPU memory and reducing the training time by one-third.

#### 4.4.4. Other Parameters Comparison

The comparative results of the above evaluation metrics are concluded in Table 6, which shows that the HRCC-Net improves the best results in terms of accuracy, sensitivity, specificity, and F1 score by 1.3%, 0.4%, 1.3%, and 1.2%, respectively, compared with the other models, which indicates that the model in this paper can obtain superior classification results. In general, the performance of a medical diagnostic system can be measured by its specificity and sensitivity. The increase in sensitivity and specificity indicates that the method can improve diagnostic efficiency while reducing the rate of misdiagnosis.

The performance of the proposed HRCC-Net is measured by introducing other technical parameters, such as FLOPs and test times. As shown in Table 7, note that for the 25,596 test images provided in the ChestX-ray14 test set, the time cost is recorded and averaged at the end of the test run. The experimental results show that the method in this paper is less computational while the average AUC value is higher compared to other networks. The computational effort is larger compared to [10]. Still, the AUC values and average test times are satisfactory, indicating that the algorithm can maintain efficient inference and diagnosis with a reasonable computational effort. In contrast, the method is valuable for improving the performance of chest disease classification on the ChestX-ray14 dataset.

### 4.5. Ablation Study

The unique feature of the proposed HRCC-Net network is the use of a multi-resolution network HRNet, CondConv module, and CA mechanism. To assess the effectiveness of each module or component in the model, ablation experiments are conducted on the ChestX-ray14 dataset. In this case, the DenseNet-121 pre-trained on ImageNet is used as the baseline network for this experiment, the last global average pool is replaced with the maximum global pool, and the last classifier is replaced with a 14-dimensional fully connected layer. The proposed model is compared with the DenseNet-121 baseline network while activating one or more modules. The results of the ablation experiments are shown in Table 8. It is observed that it is up to 4% higher than the baseline network when using only the HRNet backbone network. The high-resolution branch of the multi-resolution backbone network preserve key detailed features of the disease, and the multi-resolution fusion module provides much richer contextual information to characterize the disease in complex pathologies, so the ability to identify the disease is substantially improved. With the addition of the CondConv module, the average AUC is increased by 0.7%. This module can improve the multi-scale expression capability of the network, which can capture the lesion size adaptively and adapt to the dimensional changes of various diseases. The introduction of the CA module enables the model to obtain better performance. CA module enables the network to focus more on the focal region, concentrate more information processing on the target region, and exclude the interference of irrelevant tissues. By combining them, the average AUC score reached 84.5%, 5.2% higher than the baseline network.

#### 4.5.1. Selection of Backbone Network

**Figure 8:**
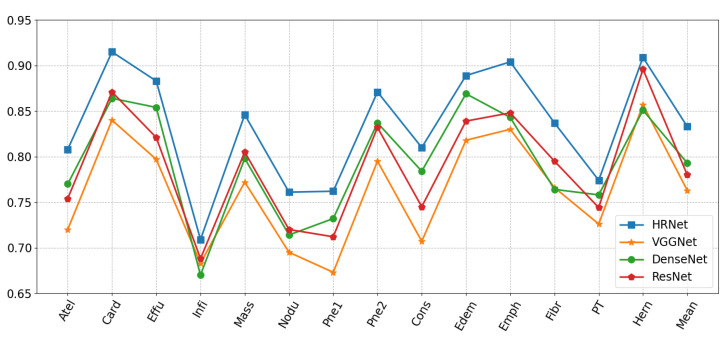
Per-class AUC of different backbone networks.

In this paper, the backbone network is critical in determining classification performance. A multi-resolution branching parallel network HRNet [8] is chosen as the backbone classification network in this study to maintain a high-resolution representation. Some current frameworks (e.g., ResNet, DenseNet) encode the input image as a low-resolution representation by concatenating high- to low-resolution convolutions. This experiment compares HRNet with the basic VGGNet-16, ResNet-101, and DenseNet-121 networks that use pre-trained weights in our experiments. For these CNN networks, transfer learning allows the extraction of as many common features as possible from a large amount of training data, thus making the learning burden of the model lighter for a specific task. For the current multi-label thoracic disease classification task, some parameters may not be suitable; the current parameters were fine-tuned to obtain better results by overlapping with all our tasks. Loading weights are pre-trained on ImageNet by freezing the weight parameters of all networks except the last fully connected layer, and modifying the classifier of the last fully connected layer by changing the classifier to a 14-dimensional fully connected layer. The results are shown in Figure 8. Showing that the above network using serial connection indeed loses some feature information during constant convolution, the HRNet network with parallel multi-resolution convolution and feature fusion units enables the critical details of the lesions to be extracted and to adequately study the complicated appearance of the disease to distinguish the lesions with different characteristics. In terms of classification results, cardiomegaly, nodule, mass, and emphysema all have significant improvement, and for some diseases with strong inter-class similarity, such as infiltration, the improvement is also obvious.

#### 4.5.2. Effectiveness of the CondConv Module

HRCC-Net uses multiple convolution kernels to extract multi-scale information from lesions to achieve a finer granularity of multiple available, receptive fields in convolution for feature extraction. The experiments are compared with a multi-resolution parallel network as a baseline to demonstrate the necessity of the CondConv module, as Table 9 shows. In this case, the Inception module, multi-branch grouped convolution, is compared with the CondConv module. The Inception module can improve model performance but is computationally expensive. In contrast, multi-branch grouped convolution is relatively lightweight but has slight model performance improvement. The CondConv module dynamically aggregates multiple parallel convolution kernels based on attention weights in a way that requires only one convolution to be computed without increasing the computational cost and adapting to variations in disease size.

#### 4.5.3. Effectiveness of the CA Module

To demonstrate the performance of coordinate attention, a series of ablation experiments are performed with a multi-resolution network as the baseline, as shown in Table 10. The importance of encoding coordinate information is understood by removing horizontal or vertical attention from coordinate attention. The model’s performance with attention along any direction is comparable to that of the model with SE attention. The SE module only considers the channel features. In chest radiograph images, due to the variations in lesion size and location, spatial features must also be considered to focus on the lesion area and identify the spatial location of the abnormal pathology. Meanwhile, the CBAM is compared with the CA module, where the former uses dual attention but tries to exploit positional information by reducing the channel dimensionality of the input data, and then uses convolution to compute spatial attention that only captures local relationships. On the other hand, CA embeds positional information into channel attention, considering both pixel and location features, which can capture the long-distance dependence of lesions. The results show that our adopted method can identify and localize the exact location of the lesion more accurately.

### 4.6. Visualized Analysis

**Figure 9:**
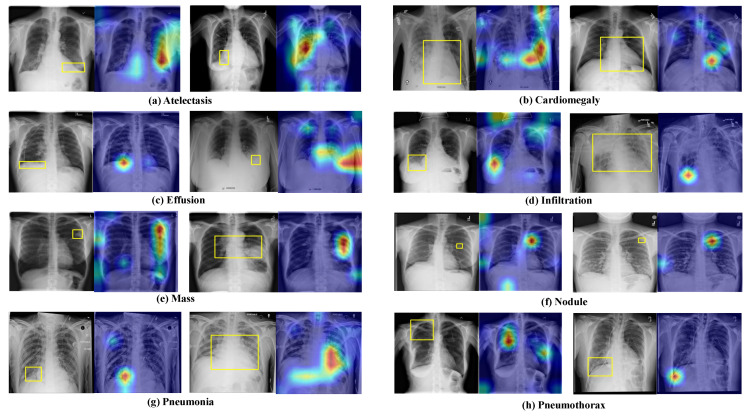
The right side of the figure shows the heat map, and the left side shows the physician-labeled map with the disease location marked by a yellow rectangular box. The figure visualizes that the network can accurately locate the diseased area, which is highly overlapping with the physician-labeled map, indicating that the effect of this network is more pronounced.

To further demonstrate the effectiveness of the HRCC-Net network, the decision process of the model is visualized by using the Gradient Weighted Class Activation Mapping (Grad-CAM) method. The generation of heat maps qualitatively analyzes whether the model is focused on the correct location and whether the learned features are rich, providing an explanatory view of the model’s effectiveness. The absolute eigenvalue of each position is first obtained from the last convolutional layer of the HRCC-Net model, and then the maximum value is calculated along the feature channel. This experiment uses 983 chest radiograph disease locations from eight diseases in the ChestX-ray14 dataset to map disease annotations. As shown in Figure 9, it can be observed that discriminative regions of the images are activated, allowing a visual assessment of the network’s ability to locate lesion locations and learn disease features. The brighter the color of each area in the heat map, the more attention the model pays to that location and the richer the features learned. The results show that the multi-scale attention module allows the model to adapt to variations in the size of pathological abnormalities, automatically focus on lesion regions, and learn abundant disease features to identify lesions accurately. More major-sized chest diseases, such as the cardiomegaly in Figure 9b and small-sized disease mass in Figure 9e, etc., can be captured automatically. For different locations of disease, such as pneumothorax, regions of hyperactivation are approximately matched to the bounding box of the pathological abnormality, indicating that the network can identify the spatial location of the pathological abnormality and accurately localize to the diseased region.

## 5. Discussion

This study proposes a multi-scale high-resolution classification network with dynamic convolution and coordinate attention (HRCC-Net). Specifically, some thoracic diseases have a significant degree of similarity in the appearance; the size of the lesions varies considerably, different conditions commonly occur in various locations of the thorax, and there are a large number of areas in the CXR images that are not unrelated to the disease. However, the existing thoracic disease classifiers based on CNN have insufficient feature extraction for thoracic lesions and lose essential details, which leads to the recognition accuracy being less than optimal. Such scale variability and location diversity make it difficult for the model to adapt to variations in the scale and location of the disease. Therefore, this study proposes a multi-scale high-resolution classification network to classify complex and diverse multi-label thorax diseases. The study is evaluated on the ChestX-ray14 dataset, and the proposed model achieves an average AUC of 0.845 in classifying 14 thoracic diseases. In addition, this study validates the performance of HRCC-Net on the CheXpert dataset, showing that the proposed solution can be extended and retrained for the classification of CXR images of various pathologies. This algorithm is generalizable and robust.

Compared with other research methods in Table 3, the network in this paper obtained satisfactory results. When using a multi-resolution backbone network, the classification results are competitive compared to other networks. This is because the network maintains the high-resolution representations of the images during feature extraction, where the high-resolution branch can extract the vital detail features of the lesions to distinguish them. The multi-resolution fusion module can acquire multiple receptive fields to obtain rich semantic information, which can identify the subtle differences of the lesions and thus improve classification accuracy. The terminal classification model HRCC-Net has higher accuracy and is adaptable to diseases of different sizes, such as cardiomegaly and pleural thickening. This is explained by the ability of the multi-scale convolution module to extract multi-scale information from each convolutional layer, adapting to variations in disease size. Meanwhile, the coordinate attention module focuses on the images’ pixel features and location information simultaneously, enabling the network to focus on the relevant regions of the disease and capture the location changes automatically. In addition, the complexity of the model is also considered, and the present model can experiment with high inference performance with lower complexity, and the multi-scale convolution module and coordinate attention module do not increase excessive computational consumption.

Moreover, the effectiveness of each module through ablation experiments. Our multi-resolution network has significantly improved classification results compared to other backbone networks. The Inception module containing multiple convolution kernels can improve the model capability but brings a large amount of computation. In contrast, multi-branch group convolution is lightweight but inferior in extracting multi-scale information. Dynamic convolution allows the network to adapt to variations in disease size without increasing the computational cost too much. The coordinate attention mechanism performs better than SE attention and CBAM attention, enabling more accurate identification and localization of the lesion’s exact location. Although our network increased the attention to spatial information while focusing on the channel information of features, it did not focus on the information on the correlation between different chest diseases.

## 6. Conclusions

This study proposes a multi-scale high-resolution classification network with dynamic convolution and coordinate attention (HRCC-Net) to classify complicated and diverse multi-label thorax diseases, capable of extracting critical detail features of lesions and capturing changes in size and location. The network consists of multiple branches from high to low resolution, which can maintain the high-resolution representations of the input image throughout the path, and the multi-resolution swapping module can obtain abundant disease features, thus extracting critical detail information for the identification of complicated thoracic diseases, especially those with similar feature presentation. Dynamic convolution is utilized in each convolutional layer to acquire multi-scale receptive fields and thus obtain multi-scale information about the illness to accommodate different scales of lesions. In addition, the coordinate attention mechanism is introduced to focus on the disease’s pixel features and location features to identify the spatial location of abnormal regions and capture changes in lesion location. The study is evaluated on the ChestX-ray14 and the CheXpert datasets, with average AUC of 0.845 and 0.913, respectively. Compared with several currently advanced methods, this study proves the competitiveness of HRCC-Net and performs a series of ablation experiments to verify the method’s effectiveness. Although the proposed method achieves high classification performance, it still has a few limitations. This paper has not attended to the information on the correlation between different thoracic diseases. Moreover, this study assumes that all labels in the ChestX-ray dataset are valid, ignoring the error rate of the labels in the dataset.

In future work, the following issues will be further studied. (1) Due to the intrinsic correlation between multiple diseases, the potential semantic relationships between diseases can be explored by introducing Transformer as a priori knowledge. (2) Resolving the uncertainty of the presence of noise labels: most of the existing classification methods ignore the problem that labels are hardly completely realistic and valid [47]. Multiple levels of weight assignment and replacement can be applied to the tags to eliminate noise and thus reduce the interference of noisy tags. (3) Processing ambiguity and uncertainty of medical images: CNN alone cannot handle the uncertainty and fuzziness present in the images, with the help of fuzzy sets, these irrelevant noises and undesired background parts can be handled during the image fusion process [48,49].

## Figures and Tables

**Table 1:** The network structure of HRCC-Net, where Transition stands for fusion module, and Concat stands for channel cascade. Each multi-scale attention module containing 4 multi-scale modules and coordinate attention.

Layer	Operation	Num_Branches	Resolution Branch	Output Size	Output_Channels
Input			224 × 224	3	
Stem	Conv3 × 3 Block Conv3 × 3 Block		1 1	112 × 112 56 × 56	64 64
Stage1	Bottleneck Block × 4 Transition1	1	1, 1/2	56 × 56 28 × 28	48 96
Stage2	Multi-Scale Attention Module × 4 Transition2	2	1, 1/2, 1/4	56 × 56 28 × 28 14 × 14	48 96 192
Stage3	Multi-Scale Attention Module × 4 Transition3	3	1, 1/2, 1/4, 1/8	56 × 56 28 × 28 14 × 14 7 × 7	48 96 192 384
Stage4	Multi-Scale Attention Module × 4 Transition4	4	1, 1/2, 1/4, 1/8	56 × 56 28 × 28 14 × 14 7 × 7	48 96 192 384
Output Head	Downsample, Contcat Downsample, Contcat Downsample, Contcat			96 192 384	
Classification	Conv1 × 1 Block Linear1 Linear2		1 × 1 1 × 1 1 × 1	2048 512 14	

**Table 2:** Selection of HRCC-Net hyperparameters.

Lr	Batch Size		Epoch
	64	128	
0.0006	0.8396	0.8348	90
0.0008	0.8415	0.8402	80
0.001	0.8408	**0.8451**	80
0.0012	0.8391	0.8432	75
0.0014	0.8346	0.8358	70

**Table 3:** Comparative results of different methods on ChestX-ray14.

Disease	[14]	[40]	[41]	[13]	[10]	[9]	[42]	[43]	[39]	Baseline	Ours
	R-50	*	R-101	D-121	D-121	D-121	*	D-121	GCN		
Atel	0.700	0.733	0.763	0.781	0.785	0.779	0.770	0.785	0.792	0.808	**0.823**
Card	0.810	0.865	0.884	0.880	0.899	0.895	0.870	0.897	0.892	**0.915**	0.908
Effu	0.758	0.806	0.816	0.829	0.835	0.836	0.830	0.837	0.840	0.883	**0.886**
Infi	0.661	0.673	0.679	0.702	0.699	0.710	0.710	0.706	**0.714**	0.709	0.711
Mass	0.693	0.718	0.801	0.834	0.838	0.834	0.830	0.834	**0.848**	0.846	**0.848**
Nodu	0.669	0.777	0.729	0.773	0.775	0.777	0.790	0.786	**0.812**	0.761	0.781
Pne1	0.658	0.684	0.710	0.729	0.738	0.737	0.720	0.730	0.733	0.762	**0.768**
Pne2	0.799	0.805	0.837	0.857	0.871	0.878	0.880	0.871	0.885	0.871	**0.897**
Cons	0.703	0.711	0.744	0.754	0.763	0.759	0.740	0.763	0.753	0.810	**0.819**
Edem	0.805	0.806	0.841	0.850	0.850	0.855	0.840	0.854	0.848	0.889	**0.897**
Emph	0.833	0.842	0.884	0.908	0.924	0.933	0.940	0.921	**0.948**	0.904	0.933
Fibr	0.786	0.743	0.801	0.830	0.831	**0.838**	0.830	0.817	0.827	0.837	0.808
PT	0.684	0.724	0.754	0.778	0.776	0.791	0.790	0.791	0.795	0.774	**0.808**
Hern	0.872	0.775	0.876	0.917	0.922	0.938	0.910	**0.943**	0.932	0.909	**0.943**
Mean	0.745	0.761	0.794	0.816	0.822	0.826	0.819	0.824	0.830	0.833	**0.845**

* The 14 pathologies are Atelectasis (Atel), Cardiomegaly (Card), Effusion (Effu), Infiltration (Infi), Mass (Mass), Nodue (Nodu), Pneumonia (Pne1), Pneumothorax (Pne2), Consolidation (Cons), Edema (Edem), Emphysema (Emph), Fibrosis (Fibr), Pleural Thickening (PT) and Hernia (Hern), respectively. * represents that the combination of ResNet and DenseNet is used in [40,42].

**Table 4:** Comparison results of HRCC-Net on CheXpert.

Policy	Method	Atelectasis	Cardiomegaly	Consolidation	Edema	Pleural Effusion	Mean
Zeros	[38]	0.811	0.840	0.932	0.929	0.931	0.889
	[44]	0.806	0.833	0.929	0.933	0.921	0.884
	[10]	0.804	0.874	0.940	0.894	0.923	0.889
	Baseline	0.796	0.830	0.928	0.897	0.925	0.875
	Ours	**0.828**	**0.882**	**0.943**	**0.937**	**0.930**	**0.904**
Ones	[38]	**0.858**	0.832	0.899	0.941	0.934	0.893
	[44]	0.825	0.855	0.937	0.930	0.923	0.894
	[10]	0.847	0.868	0.923	0.924	0.926	0.898
	[43]	0.848	0.865	0.908	0.912	0.940	0.895
	Baseline	0.770	0.849	0.942	0.906	0.928	0.879
	Ours	0.847	**0.891**	**0.945**	**0.940**	**0.943**	**0.913**

**Table 5:** Computational consumption of HRCC-Net.

Model	Parameters/M	FLOPs/G	Batch Size (Times/h)	
			64	128
Baseline	20.9	9.71	-	-
Baseline+CondConv	23.1	10.87	-	-
Baseline+CondConv+CA	24.3	11.25	-	-
HRCC-Net	24.3	11.25	22	7.1

**Table 6:** Comparison of other evaluation metrics on the ChestX-Ray14 dataset.

Method	Accuracy	Sensitivity	Specificity	F1
[14]	75.6	73.4	76.1	72.9
[45]	75.9	73.8	76.4	73.3
[46]	76.7	74.3	76.9	73.7
[44]	77.2	74.9	77.3	74.1
[39]	77.5	75.3	77.6	74.3
Ours	78.8	75.7	78.9	75.5

**Table 7:** Other technical parameters comparison.

Model	FLOPs/G	Times/S	Average AUC
[14]	21.47	**0.031**	0.745
[45]	14.97	0.035	0.807
[10]	**2.96**	0.350	0.822
[20]	15.03	0.045	0.823
[46]	34.96	0.094	0.824
[9]	-	0.100	0.826
[39]	17.74	0.059	0.830
Ours	11.25	0.041	**0.845**

**Table 8:** Ablation experiments on the ChestX-ray14 dataset.

HRNet	CondConv	CA	Mean
			0.793
✓			**0.833**
✓	✓		0.840
✓		✓	0.839
✓	✓	✓	**0.845**

**Table 9:** Comparative experiments of the CondConv module.

Setting	Average AUC	Parameters/M
Baseline	0.8330	20.9
+Inception	0.8382	27.0
+GroupConv	0.8348	21.8
+Condconv	0.840	23.1

**Table 10:** Comparative experiments of the CA module.

Setting	Average AUC	Parameters/M
Baseline	0.8330	20.9
+SE	0.8344	21.46
+X Attention	0.8344	21.46
+Y Attention	0.8345	21.46
+CBAM	0.8358	21.88
+CA	0.8372	22

## Data Availability

Data will be made available.
